# Good Quality of Life in Former Buruli Ulcer Patients with Small Lesions: Long-Term Follow-up of the BURULICO Trial

**DOI:** 10.1371/journal.pntd.0002964

**Published:** 2014-07-10

**Authors:** Sandor Klis, Adelita Ranchor, Richard O. Phillips, Kabiru M. Abass, Wilson Tuah, Susanne Loth, Kristien Velding, Tjip S. van der Werf, Ymkje Stienstra

**Affiliations:** 1 Department of Internal Medicine, Infectious Disease Service, University Medical Center Groningen, Groningen University, Gronigen, the Netherlands; 2 Department of Health Sciences, Section Health Psychology, University Medical Center Groningen, Groningen University, Gronigen, the Netherlands; 3 Department of Internal Medicine, Komfo Anokye Teaching Hospital, Kumasi, Ghana; 4 Agogo Presbyterian Hospital, Agogo, Ghana; 5 Nkawie-Toase Government Hospital, Nkawie, Ghana; 6 Department of Pulmonary Medicine and Tuberculosis, University Medical Center Groningen, Groningen, the Netherlands; Fondation Raoul Follereau, France

## Abstract

**Background:**

Buruli Ulcer is a tropical skin disease caused by *Mycobacterium ulcerans*, which, due to scarring and contractures can lead to stigma and functional limitations. However, recent advances in treatment, combined with increased public health efforts have the potential to significantly improve disease outcome.

**Objectives:**

To study the Quality of Life (QoL) of former Buruli Ulcer patients who, in the context of a randomized controlled trial, reported early with small lesions (cross-sectional diameter <10 cm), and received a full course of antibiotic treatment.

**Methods:**

127 Participants of the BURULICO drug trial in Ghana were revisited. All former patients aged 16 or older completed the Dermatology Life Quality Index (DLQI) and the abbreviated World Health Organization Quality of Life scale (WHOQOL-BREF). The WHOQOL-BREF was also administered to 82 matched healthy controls. Those younger than 16 completed the Childrens' Dermatology Life Quality Index (CDLQI) only.

**Results:**

The median (Inter Quartile Range) score on the DLQI was 0 (0–4), indicating good QoL. 85% of former patients indicated no effect, or only a small effect of the disease on their current life. Former patients also indicated good QoL on the physical and psychological domains of the WHOQOL-BREF, and scored significantly higher than healthy controls on these domains. There was a weak correlation between the DLQI and scar size (*ρ* = 0.32; *p*<0.001).

**Conclusions:**

BU patients who report early with small lesions and receive 8 weeks of antimicrobial therapy have a good QoL at long-term follow-up. These findings contrast with the debilitating sequelae often reported in BU, and highlight the importance of early case detection.

## Author Summary

Buruli ulcer is an infectious skin disease, mainly occurring in West Africa. It usually starts with a small nodule that over the course of weeks progresses into an ulcer. Effective treatment with antibiotics is available, but patients often report to the hospital late, and there is a serious risk of scarring, contractures and functional limitations of the affected body parts. Although it is often reported that Buruli ulcers can have serious sequelae, the quality of life of former Buruli ulcer patients has not been studied before. In this study we assessed the quality of life of healed Buruli ulcer patients that took part in a drug trial between 2006 and 2009, which only included small and early lesions. On follow-up, we found that most scars were small and functional limitations were rare, and that both general and skin specific Quality of Life was good. Our results demonstrate the potential of the combination of early detection and proper antibiotic treatment of Buruli ulcer and hence stress the importance of public health efforts aimed at diagnosing the disease in its early stage, and providing standardized treatment in endemic areas.

## Introduction

Buruli ulcer (BU) is listed by the World Health Organization (WHO) as a neglected tropical disease, caused by infection with *Mycobacterium ulcerans*. Although the disease has been reported from as many as 30 countries around the world, it is currently most common in West and Central Africa, and it predominantly affects the rural poor. Typically, the disease starts with a small, painless nodule that progresses into a large necrotizing ulcer over the course of several weeks. After treatment, although the ulcer usually heals, there is a high risk of significant scarring, contractures and functional limitations [1]–[3].

In the socio-economic context of rural Africa, functional limitations and stigmatizing scars can have severe consequences. In a study of 638 former BU patients in Ghana and Benin, 57% appeared to have a functional limitation, and school dropout, financial difficulties and job loss were frequent consequences of the disease [2]. People in endemic communities sometimes perceive the disease to be caused by a curse or witchcraft, and the resulting stigma can cause social isolation and problems with finding work or a spouse [4]–[6]. Although the treatment for BU is free of charge, the costs associated with hospitalization can be devastating for the household economy and frequently cause family members to cease providing financial and social support to patients [7].

However, over the past decade, the main mode of treatment has shifted from surgery to antibiotics, with high rates of cure [8]. In addition, significant public health efforts have been directed at detecting BU at an early stage and educating affected communities about the disease. Conceptually, both factors combined should reduce scarring, contractures, and stigma, and improve the subsequent quality of life (QoL) of former patients.

To our knowledge, the QoL of former BU patients has not yet been studied. In leprosy and podoconiosis, similarly deforming and stigmatizing skin conditions occurring in the tropics, several studies on QoL have been conducted in Bangladesh, Brazil, China, Ethiopia, Ghana, and India [9]–[15]. In general, these studies show that patients report a substantially lower QoL than controls [9]–[11]. There appears to be some relationship between QoL and the severity of the disease in terms of number of lesions, functional limitations, stigma, and deformities [11]–[15]. Studies from Ethiopia in podoconiosis patients, a disabling and stigmatizing geochemical elephantiasis of the foot, found that a dermatology-specific QoL instrument was valid and sensitive to therapeutic change [16], [17].

These studies demonstrate that measuring QoL with standardized questionnaires in rural Africans suffering from skin diseases is feasible. Moreover, as both leprosy and podoconiosis were shown to have a significant impact on QoL they show that measuring QoL in these populations is warranted. In the current study, we report on the disease-specific and general QoL of former BU patients who previously presented with small, early ulcers and who were treated with a full course of 8 weeks of antibiotics.

## Methods

### Sample size and patient recruitment

Our study subjects were former BU patients that had earlier participated in the BURULICO trial, conducted between 2006 and 2009 in Ghana, registered with number NCT00321178 at clinicaltrials.gov. For that trial, patients aged 5 years or older, clinically diagnosed with early (duration <6 months), limited (cross-sectional diameter of induration <10 cm, including plaques and oedemas) *M. ulcerans* infection were included, and randomized to receive either 8 weeks of streptomycin at 15 mg/kg daily (max 1000 mg daily) and rifampicin at 10 mg/kg daily (max 600 mg daily), or 4 weeks of streptomycin and rifampicin, followed by 4 weeks of rifampicin and clarithromycin at 7.5 mg/kg daily. The rate of healing did not differ between both arms. Patients had a median age of 12 and 30% were male [8].

For the present follow-up study, participants were traced between June and November 2012 by visiting their last known village or through telephone contact if available. If the former patient was no longer living at the last known village, neighbors, relatives, and community leaders were asked for additional information. When a former patient was located, he or she was informed about the study, given time to consider participation, and asked for consent.

### Questionnaires

The Cardiff Dermatologic Life Quality Index (DLQI) and its pediatric adaptation the Childrens Dermatologic Life Quality Index (CDLQI) are dermatology-specific QoL instruments [18], [19]. Both contain 10 questions with scores on a question ranging from 0 to 3. The total score is calculated by summing the score of each question resulting in a maximum of 30 and a minimum of 0. The higher the score, the more QoL is impaired. In addition, to facilitate interpretation, banding scores are available for both the DLQI and CDLQI, with a score of 0 or 1 indicating no effect, a score between 2 and 5 a small effect, a score between 6 and 10 a moderate effect, a score between 11 and 20 a very large effect, and a score between 21 and 30 an extremely large effect on the patients' life. The DLQI was designed for patients aged 16 or above, and the CDLQI for those aged between 4 and 16. Both the DLQI and CDLQI have been extensively validated, but only the DLQI has been used in low and middle Income countries, including sub-Sahara Africa [9], [16], [20], [21]. Both the DLQI and CDLQI were translated into the local language, Twii, according to the instructions of the authors of the original questionnaires. Two independent translators separately translated the questionnaire from English into Twii, and discussed their translations to arrive at a single Twii translation. A third and fourth translator then independently translated the questionnaires back into English. Next, the back-translations were reviewed by the original authors of the questionnaire. After initial comments and a subsequent cycle of translation and back-translation, the questionnaire authors approved this back-translation for further use. Finally the agreed translation was pretested in a group of 8 former BU patients that did not participate in the BURULICO trial, asking them about the clarity, understandability and wording of the questions. In this pretest no further issues arose.

The WHO Quality of Life-BREF (WHOQOL-BREF), is an international cross-culturally comparable generic QoL assessment instrument [22]. It assesses the individuals' perceptions in the context of their culture and value systems, and their personal goals, standards and concerns. It comprises 26 items, which measure 4 domains: physical health, psychological health, social relationships, and environment (satisfaction with one's living conditions). In addition, two questions measure general health and general QoL. The score on each question ranges between 1 and 5, and for each domain a total score is computed that ranges between 20 and 100, with a higher score indicating a better QoL. No specific age range is given for the WHOQOL-BREF, but it was designed for adults. The WHOQOL-BREF was also translated into Twii according to the procedure outlined above for the DLQI and CDLQI, with the exception that the back translations were not reviewed by the authors of the questionnaire but by the study team. Effect sizes of the difference in WHOQOL-BREF domains between former patients and controls were calculated using the formula *z*/√N, where *z* is the *z*-score of the U statistic, and N is the total sample size.

The Buruli Ulcer Functional Limitation Score (BUFLS) is a questionnaire that consists of questions related to 19 common daily activities of people living in endemic areas [2], [23]. Each item is scored between 0 and 2, with 0 indicating no difficulties in performing the activity compared to age- and sex matched community members, 1 indicating difficulties performing the activity, and 2 indicating that the former patient is unable to carry out the activity at all. In the calculation of the individual functional limitation score, the sum is divided by the maximum possible score for that individual, multiplied by 100%. A higher score therefore indicates more functional limitations, with a range between 0% and 100%. A score cannot be calculated if more than 6 items are not applicable.

### Procedures

Former patients currently aged 16 or above were administered the WHOQOL-BREF, DLQI and BUFLS questionnaires, and former patients currently aged below 16 were administered the CDLQI and BUFLS only. The WHOQOL-BREF was also administered to 82 age, sex, and occupation (farming, schooling or other) matched healthy controls that lived in the same villages as the former BU patients on separate visits. All potential controls were asked whether they were currently sick or injured, and whether they were on any medication; they could only participate if they answered both questions with no. Due to high rates of illiteracy among the participants, all questionnaires were administered orally in a quiet private place by three trained local hospital staff members. In addition all patients were asked about the presence of pain (Yes/No) and itch (Yes/No), marital status, level of education and employment. Also, for all former patients the scar size was determined by tracing it on a transparent sheet, scanning it, and measuring the surface with ImageJ software.

### Ethics

The study protocol was approved by the Committee on Human Research, Publication, and Ethics of the Kwame Nkrumah University of Science and Technology and the Komfo Anokye Teaching Hospital, Kumasi (reference number CHRPE/AP/133/12). Written and verbal informed consent or assent was obtained from all participants aged ≥12 years, and consent from parents, or legal representatives of participants aged ≤18 years.

## Results

### Patient population

127 individuals (84%) of the 151 former participants of the BURULICO trial were located for follow up, and none declined to participate. The median duration between drug treatment and enrolment in the current study was 5 years. Although the trial had taken place in the Ashanti region of Ghana, many former patients had moved away from the study site, and patients were retrieved in 9 of 10 Ghanaian regions, including the three Northern regions more than 700 kilometers and approximately 10 hours by road from the original study site. 68% of the retrieved former patients were female, and the median age at follow-up was 18 years. 71 former patients were age-eligible, and completed the DLQI, but due to an error the WHOQOL-BREF was not administered to 4 former patients, so data on this questionnaire were only available for 67 former patients. The remaining 56 former patients were administered the CDLQI only. There was no missing data on the DLQI and CDLQI, and a maximum of 2 missing answers per QoL domain on the WHOQOL-BREF, meaning that domain scores could be calculated for every subject. Item 21 of the WHOQOL-BREF, “how satisfied are you with your sex life?”, caused considerable confusion among many former patients who were not married. Even after repeated explanation that one could still have an opinion about one's sex life if you are not married (i.e. be dissatisfied with it), it was poorly understood and hence left open on 20 of the 67 questionnaires. Due to a printing error, the BUFLS was not administered to one former patient, but meaningful scores (i.e. less than 6 items not applicable) could be computed for all other patients.

Of the 24 former patients not retrieved, 4 were already lost to follow-up during the BURULICO trial, 3 had moved abroad, 2 had deceased, and the fate of the remaining 15 was unknown. The former patients that were lost to follow-up did not differ significantly from those that were retrieved in terms of age, gender or treatment arm.

### Quality of Life scores and patient characteristics

**Figure 1:**
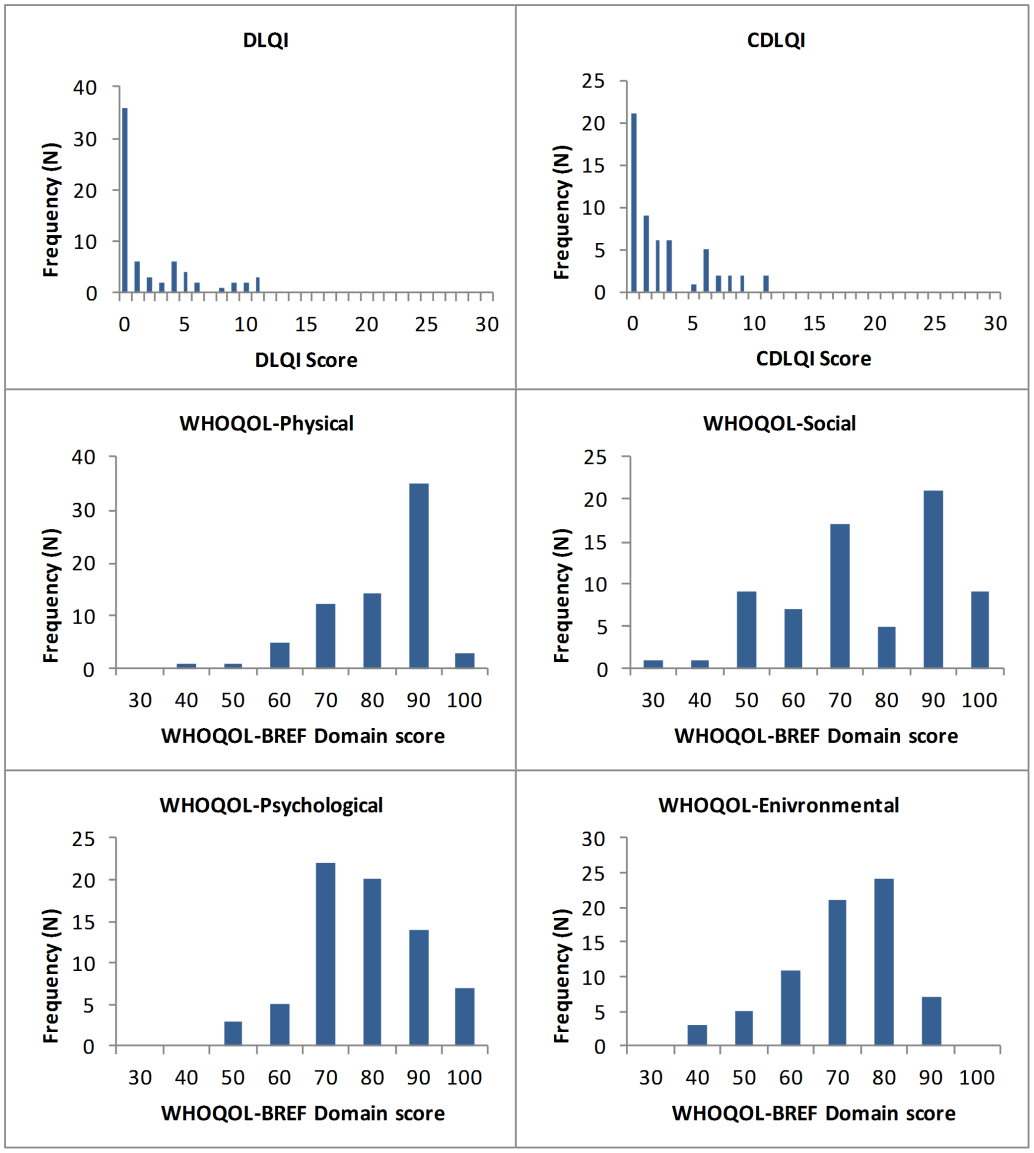
Frequency distributions of the DLQI, CDLQI, and WHOQOL-BREF scores. Theoretical range (C)DLQI = 0–30, WHOQOL = 20–100. DLQI = Dermatology Life Quality Index, CDLQI = Childrens Dermatology Life Quality Index.

Scores, range, and reliability for the DLQI, CDLQI, and WHOQOL-BREF subscales are shown in Table 1. The frequency distributions of the QoL scales are shown in Figure 1. Upon inspection, it appears that the psychological and environmental subscales of the WHOQOL-BREF were normally distributed. The distribution of the DLQI and CDLQI is skewed to the left. The banding scores for the DLQI and CDLQI are shown in Table 2.

**Table 1:** Scale descriptives for the DLQI, CDLQI and WHOQOL-BREF subscales.

Scale	Mean (SD)	Median (IQR)	Range	Cronbach's α
*DLQI*	2.3 (3.4)	0 (0–4)	0–11	.78
*CDLQI*	2.6 (3.2)	1 (0–4.5)	0–11	.57
*WHOQOL-BREF Physical*	75.9 (12.0)	80 (69–86)	37–94	.63
*WHOQOL-BREF Psychological*	70.9 (12.1)	70 (60–80)	40–97	.62
*WHOQOL-BREF Social*	67.9 (18.0)	68 (53–80)	20–100	.55
*WHOQOL-BREF Environmental*	65.1 (11.6)	68 (58–73)	35–88	.66

N = 71 for the DLQI, N = 67 for the WHOQOL-BREF, and N = 56 for the CDLQI. SD = standard deviation, IQR = interquartile range. DLQI = Dermatology Life Quality Index, CDLQI = Childrens Dermatology Life Quality Index, WHOQOL-BREF = abbreviated World Health Organisation Quality of Life instrument.

**Table 2:** Banding scores of the DLQI and CDLQI.

Effect of scar or contracture on patients' life	DLQI (N = 67)	CDLQI (N = 56)
*No effect*	63%	54%
*Small effect*	22%	32%
*Moderate effect*	10%	14%
*Very large effect*	5%	0%
*Extremely large effect*	0%	0%

DLQI = Dermatology Life Quality Index, CDLQI = Childrens Dermatology Life Quality Index.

Former patients had a median (IQR) scar size of 4.6 (1.4–13.5) cm^2^, and a median (IQR) BUFLS score of 2.6% (0%–13.3%). Only 4 (3%) former patients were unemployed, all others were either studying or working. Only 52% of former patients were married, but those not married were significantly younger (*t* = −2.31; *p* = 0.024). The scar was itchy in 26% and painful in 9% of former patients.

### Associations between Quality of Life instruments and patient characteristics

Spearman's Rho correlations between the DLQI, CDLQI and WHOQOL-BREF subscales and other continuous variables are shown in Table 3. Age was not significantly related to any of the QoL scales. Scar size correlated significantly with the DLQI (*ρ* = 0.32; *p*<0.01). The BUFLS correlated significantly with the environmental subscale of the WHOQOL-BREF (*ρ* = −0.24; *p*<0.05).

**Table 3:** Spearman's Rho correlations between QoL scales and other continuous variables.

	*DLQI*	*CDLQI*	*WHOQOL-BREF Phys*	*WHOQOL-BREF Psy*	*WHOQOL-BREF Soc*	*WHOQOL-BREF Env*
*BUFLS*	.15	.11	−.14	−.10	−.02	−.24*
*Age*	−.01	−.01	−.16	−.20	−.06	−.08
*Scar size*	.32**	.12	−.06	−.03	.09	.14

* = p<0.05,

** = p<0.01.

DLQI = Dermatology Life Quality Index, CDLQI = Childrens Dermatology Life Quality Index, WHOQOL-BREF = abbreviated World Health Organisation Quality of Life instrument, BUFLS = Buruli ulcer Functional Limitation Score.

Relationships between the QoL instruments and discrete variables were tested through either student T-tests or Mann-Whitney U tests depending on whether the scales were normally distributed. The QoL of women did not differ from men on any of the scales or subscales. Farmers, scored significantly higher on the social domain of QoL compared to other professions (*U* = 435; *p* = 0.050). Those who were married also scored significantly higher on the social domain than those who were single (*U* = 375; *p* = 0.019). Those who were ethnically Akan scored significantly higher on the DLQI (*U* = 332; *p* = 0.026), indicating lower QoL, and significantly lower on the physical domain of QoL (*U* = 292; *p* = 0.003), again indicating lower QoL than those from the northern tribes. None of the QoL scores differed between those that did or did not report painful or itchy scars.

The DLQI correlated significantly with the physical (*ρ* = −0.54; *p*<0.001) and psychological (*ρ* = −0.34; *p*<0.001) subscales of the WHOQOL-BREF, but did not correlate at all with the social (*ρ* = 0.00; *p* = 0.99) and environmental (*ρ* = −0.01; *p* = 0.98) subscales.

### Quality of life of former BU patients compared to healthy controls

**Figure 2:**
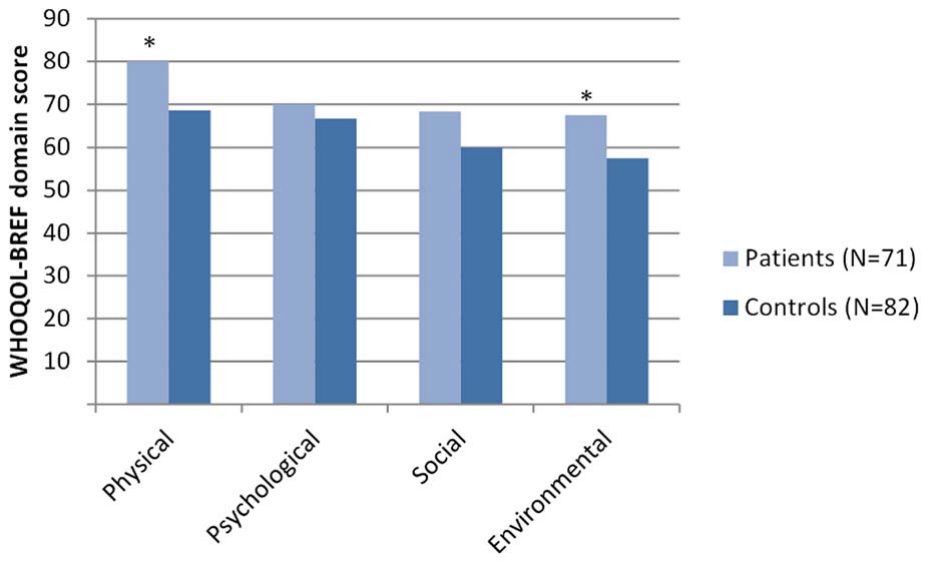
Median scores (theoretical range 20–100) on the WHOQOL-BREF domains of former patients and healthy controls. Differences between the two groups were tested with Mann-Whitney U test. * = p<0.01, others ns. WHOQOL-BREF = abbreviated World Health Organisation Quality of Life instrument.

Scores on the WHOQOL-BREF domains of patients and healthy controls are shown in Figure 2. Former BU patients scored significantly higher than controls on the physical and environmental domains of the WHOQOL-BREF by Mann-Whitney U tests. The effect sizes for the four domains were as follows: physical 0.29, environmental 0.41, psychological 0.10, and social 0.14.

## Discussion

This is the first study to address QoL in former BU patients. The former BU patients with small lesions that we studied appeared to have a good QoL. When measured with the DLQI, a disease specific questionnaire, 85% of patients indicated no or a small effect of the disease on their current life. The median score found was 0 compared to 13 in active and 3 in healed podoconiosis patients in Ethiopia [16], and median scores between 2 and 12 in patients with various active skin diseases in South Africa [21]. In the current study, scores on a measure of general QoL were high, especially in the physical domain of QoL, but – likely owing to the poor rural setting – lower on the environmental domain, which relates to issues such as access to healthcare and transportation, and financial resources. In addition, former patients indicated an equal or better QoL compared to healthy controls in all four domains measured.

As their scars and functional limitations were limited, it could be expected that former patients would not differ much from controls in QoL. However, it is surprising that they would report a higher physical and environmental QoL, with small to medium effect sizes. As controls were matched to former patients on age, gender, village and occupation, it is difficult to explain these findings on the basis of social or economic factors. Perhaps having been confronted with BU and the prospect of scarring and disability, made patients adjust their internal standards of what constitutes good physical functioning. This, in turn, would cause them to appreciate the preserved physical functioning they have today, a phenomenon known as response shift [24]. One possible explanation for indicating a better environmental QoL could be their positive experience with the healthcare system for the treatment of their BU, which is free of charge and relatively well-organized in the study regions. However, it is also possible that the interviewers were viewed by former patients as representatives of the hospitals where they were treated, causing them to answer in a more socially desirable way than controls. Another possible explanation is that we followed-up a group who presented early, in contrast with most BU patients, and that this was a consequence of this group having more access to healthcare and other resources, i.e. through being more educated or living less isolated, and hence a better baseline QoL than the average community member.

The good QoL of former patients with small lesions found in this study corresponded with the more physical measures of BU sequelae. Patients had a relatively small median scar size. In addition their level of functional limitations as measured by the BUFLS was low, with a median score of 2.6%, compared to earlier scores of 5.3% in a large cohort of BU patients with varying lesion sizes that was treated outside of any study context [2], and 16% in patients who had only received surgical or traditional treatment [25].

The DLQI had a Cronbach's alpha of 0.78 indicating satisfactory internal consistency [26]. In addition, it was significantly related to scar size, which can be seen as a proxy measure of disease severity, and to both the psychological and physical QoL domains of the WHOQOL-BREF in the expected directions. Similar to two previous studies with the DLQI in Sub-Saharan Africa, we did not find DLQI scores to be related to gender [16], [21]. Together, these findings suggest that the DLQI is a valid instrument for measuring disease-specific QoL in BU patients aged 16 and above, although further research in larger samples, including larger lesions is needed. In children, the CDLQI had a low Cronbach's alpha, and was not related to any of the background variables, which makes it difficult to ascertain its validity. The Cronbach's alphas for the WHOQOL-BREF subscales were all below 0.7, indicating questionable internal consistency. The social domain of QoL was related to being married and working as a farmer, and the physical and psychological domains were related to disease specific QoL in the expected directions. Former patients indicated a higher QoL on all 4 domains of the WHOQOL-BREF than controls, which is rather unexpected. In addition, item 21, satisfaction with ones sex life, was poorly understood by those who were not married, and was left open by a considerable proportion/number of former patients. Overall, these data do not clearly establish the validity of the WHOQOL-BREF for measuring general QoL in former BU patients.

The present study suffered from several limitations. First the sample size was relatively small, with only 67 adults completing both adult QoL instruments. As the cohort under study was predetermined by the BURULICO trial, we were not able to increase the sample size. This could have left the study underpowered to pick up associations between the questionnaires and background variables such as gender. However, several significant associations were indeed found. In addition, we were not able to recruit healthy controls for the DLQI and CDLQI as these are disease specific questionnaires, and thus had to rely on the earlier proposed norm scores, but the very low scores on both instruments are likely to reflect good QoL. Finally, we did not study QoL in former BU patients that were treated under normal service conditions. These patients would have likely had larger lesions and might have reported a lower QoL, and data on these patients could have helped to establish the validity of the questionnaires, although in our sample the DLQI already correlated significantly with scar size.

In this study, we show that in BU patients who reported early with small lesions and received either 8 weeks of streptomycin and rifampicin or 4 weeks of streptomycin and rifampicin followed by 4 weeks of clarithromycin and rifampicin, scars were small, functional limitations were uncommon and long term QoL was preserved. These findings contrast the debilitating sequelae often reported in BU, highlighting the importance of active case finding and antimicrobial treatment of BU lesions. The Twii version of the DLQI appeared to be a valid instrument to measure disease specific QoL in BU patients aged 16 or older, and was quick and easy to administer under field conditions. For children, the Twii version of the CDLQI appeared to be a less valid instrument in this population, though alternatives are lacking. The validity of the WHOQOL-BREF for measuring general QoL in former BU patients is questionable. Future studies on QoL in BU should attempt to include patients with active BU, and should include patients with larger lesions. In addition they should include alternative questionnaires for measuring general QoL in BU, such as the SF-36, or limit themselves to disease-specific QoL using the DLQI.

## Supporting Information

Checklist S1 STROBE checklist.
(DOC)
