# Supplementary material for: Good Quality of Life in Former Buruli Ulcer Patients with Small Lesions: Long-Term Follow-up of the BURULICO Trial
Source: PLoS Negl Trop Dis. 2014 Jul 10;8(7):e2964. doi: 10.1371/journal.pntd.0002964 (PMC4091870; doi:10.1371/journal.pntd.0002964)
Supplement: Checklist S1 — STROBE checklist. (DOC) [file pntd.0002964.s001.doc]

STROBE Statement—Checklist of items that should be included in reports of ***cross-sectional studies***

|  | Item No | Recommendation |
| --- | --- | --- |
| **Title and abstract** | 1 | **Line 1** (*a*) Indicate the study’s design with a commonly used term in the title or the abstract |
|  |  | **Line 16 through 36** (*b*) Provide in the abstract an informative and balanced summary of what was done and what was found |
| Introduction | | |
| Background/rationale | 2 | **Line 53 through 86** Explain the scientific background and rationale for the investigation being reported |
| Objectives | 3 | **Lines 70 through 72 and 83 through 86** State specific objectives, including any prespecified hypotheses |
| Methods | | |
| Study design | 4 | **Lines 89 through 102** Present key elements of study design early in the paper |
| Setting | 5 | **Lines 89 through 102** Describe the setting, locations, and relevant dates, including periods of recruitment, exposure, follow-up, and data collection |
| Participants | 6 | **Lines 89 through 93** (*a*) Give the eligibility criteria, and the sources and methods of selection of participants |
| Variables | 7 | **Lines 103 through 156** Clearly define all outcomes, exposures, predictors, potential confounders, and effect modifiers. Give diagnostic criteria, if applicable |
| Data sources/ measurement | 8* | **Lines 103 through 156**For each variable of interest, give sources of data and details of methods of assessment (measurement). Describe comparability of assessment methods if there is more than one group |
| Bias | 9 | **Lines 148 through 150, 152 through 153, 183 through 185** Describe any efforts to address potential sources of bias |
| Study size | 10 | **Lines 89 through 91 and 165 through 166** Explain how the study size was arrived at |
| Quantitative variables | 11 | **Lines 198 through 218** Explain how quantitative variables were handled in the analyses. If applicable, describe which groupings were chosen and why |
| Statistical methods | 12 | **Lines 198 through 218** (*a*) Describe all statistical methods, including those used to control for confounding |
|  |  | **N/A** (*b*) Describe any methods used to examine subgroups and interactions |
|  |  | **Lines 171 through 181** (*c*) Explain how missing data were addressed |
|  |  | **N/A** (*d*) If applicable, describe analytical methods taking account of sampling strategy |
|  |  | **N/A** (*e*) Describe any sensitivity analyses |
| Results | | |
| Participants | 13* | **Lines 165 through 185** (a) Report numbers of individuals at each stage of study—eg numbers potentially eligible, examined for eligibility, confirmed eligible, included in the study, completing follow-up, and analysed |
|  |  | **Lines 165 through 185** (b) Give reasons for non-participation at each stage |
|  |  | **N/A** (c) Consider use of a flow diagram |
| Descriptive data | 14* | **Lines 170 through 171** (a) Give characteristics of study participants (eg demographic, clinical, social) and information on exposures and potential confounders |
|  |  | **Lines 171 through 181** (b) Indicate number of participants with missing data for each variable of interest |
| Outcome data | 15* | **Lines 187 through 188** Report numbers of outcome events or summary measures |
| Main results | 16 | **Lines 187 through 188** (*a*) Give unadjusted estimates and, if applicable, confounder-adjusted estimates and their precision (eg, 95% confidence interval). Make clear which confounders were adjusted for and why they were included |
|  |  | **N/A** (*b*) Report category boundaries when continuous variables were categorized |
|  |  | **N/A** (*c*) If relevant, consider translating estimates of relative risk into absolute risk for a meaningful time period |
| Other analyses | 17 | **N/A** Report other analyses done—eg analyses of subgroups and interactions, and sensitivity analyses |
| Discussion | | |
| Key results | 18 | **Lines 220 through 228** Summarise key results with reference to study objectives |
| Limitations | 19 | **Lines 267 through 277** Discuss limitations of the study, taking into account sources of potential bias or imprecision. Discuss both direction and magnitude of any potential bias |
| Interpretation | 20 | **Lines 278 through 287** Give a cautious overall interpretation of results considering objectives, limitations, multiplicity of analyses, results from similar studies, and other relevant evidence |
| Generalisability | 21 | **Lines 274 through 277** Discuss the generalisability (external validity) of the study results |
| Other information | | |
| Funding | 22 | **On submission site** Give the source of funding and the role of the funders for the present study and, if applicable, for the original study on which the present article is based |

*Give information separately for exposed and unexposed groups.

**Note:** An Explanation and Elaboration article discusses each checklist item and gives methodological background and published examples of transparent reporting. The STROBE checklist is best used in conjunction with this article (freely available on the Web sites of PLoS Medicine at http://www.plosmedicine.org/, Annals of Internal Medicine at http://www.annals.org/, and Epidemiology at http://www.epidem.com/). Information on the STROBE Initiative is available at www.strobe-statement.org.
